# Machine Learning-Based Multi-Parameter Fusion Calibration for Composite Electrode FDR Soil Moisture Measurement

**DOI:** 10.3390/s26216956

**Published:** 2026-11-03

**Authors:** Yu Xu, Xizheng Li, Yinghao Song, Kun Zhang, Yuyang Liu, Yue Sun

**Affiliations:** 1 College of Physics and Electronic Engineering, Xinyang Normal University, Xinyang 464300, China; 19513579197@163.com (Y.S.); zk@xynu.edu.cn (K.Z.); yuyangliu@xynu.edu.cn (Y.L.); cathy_sy@126.com (Y.S.); 2 Xinyang Gumai Optronics Co., Ltd., Xinyang 464100, China

**Keywords:** frequency domain reflectometry, soil moisture, three-parameter calibration, time division multiplexed, agricultural soil–water monitoring

## Abstract

Reliable in situ soil moisture measurements provide essential information for irrigation scheduling and agricultural soil–water management. Among the available measurement techniques, capacitive soil moisture sensors based on frequency domain reflectometry (FDR) are widely used because of their high measurement accuracy and suitability for in situ monitoring. However, the resonant frequency of an FDR capacitive soil moisture sensor is affected simultaneously by temperature, soil electrical conductivity (EC), and water content conditions, and independent fixed coefficient corrections cannot adequately describe this coupled relationship. To address this problem, we developed a time division multiplexed three-parameter FDR sensor that uses a composite electrode to sequentially acquire a water content-related resonant frequency, a conductivity-sensitive voltage, and a temperature-sensitive voltage at the same sensing location. A joint three-parameter calibration model was then established to map the three outputs to the reference volumetric water content determined by oven drying. Comparison of five candidate calibration models showed that the backpropagation neural network (BPNN) provided the best overall performance on the development data. A paired 10-seed analysis showed no stable predictive advantage for WOA initialization over standard BPNN, so WOA was retained only as a secondary robustness analysis. After the WOA-BPNN calibration model was fixed and deployed on the embedded platform, repeat measurements under 72 temperature–salinity–water content conditions within the same prepared sandy loam system yielded an RMSE of 2.086 percentage points. Within the tested calibration domain, the coordinated three-parameter hardware and joint calibration model reduced water content errors associated with temperature and salinity variation. These repeat measurements were conducted in the same prepared sandy loam system and, therefore, do not demonstrate generalization across other soil textures, field sites, independently prepared sample batches, or seasons.

## 1. Introduction

Reliable in situ soil moisture measurements support irrigation scheduling and soil–water management in crop production [1,2,3]. Agricultural applications require sensors that remain accurate as soil and environmental conditions vary [3,4,5]. Frequency domain reflectometry (FDR) estimates water content from resonant frequency changes associated with the soil dielectric state. Its rapid response and ease of embedded integration support point-scale monitoring [6,7], but temperature, electrical conductivity, and other factors can shift sensor output away from the true water content [8,9,10,11,12,13,14].

Existing approaches can be distinguished by what is measured and how interference is handled. Empirical or polynomial models have been used to correct temperature and/or salinity effects [15,16,17]. Machine learning models can represent nonlinear responses [18,19,20,21,22,23,24,25,26], and soil moisture calibrations have used temperature, electrical conductivity, or both as auxiliary inputs [20,25,26]; for example, Moghadas and Badorreck used an artificial neural network with electrical conductivity and temperature to estimate soil moisture [26]. Integrated multiparameter sensors have combined capacitance- and conductivity-sensitive structures [17], while a reconfigurable PCB sensor switched a shared structure between moisture and temperature measurements [27]. In the studies reviewed here, these designs either addressed only part of the temperature–salinity interference problem or did not jointly calibrate co-located moisture-, conductivity-, and temperature-sensitive responses. Thus, neither multiparameter sensing nor machine learning calibration is claimed to be new here.

Soil is heterogeneous, so spatially separated sensing elements may not represent the same local medium [28,29,30,31]. A sensor that outputs only resonant frequency also provides no direct observation of the conductive and temperature states at the time of acquisition. This study, therefore, uses a switching network to obtain three raw responses sequentially from one composite electrode assembly: a water content-related resonant frequency, a conductivity-sensitive voltage, and a temperature-sensitive voltage. The channels have spatially overlapping rather than identical effective support regions, but their co-location reduces spatial mismatch relative to separate probes and provides condition-related inputs for joint calibration.

The present design also has explicit continuity with our prior FDR work [6,7]. The B-C interdigital geometry and the MC1648-based FDR signal-conditioning chain were adapted from those studies, and the CSF11-60 model had previously been used as a comparator [6]. These inherited components are not treated as new contributions. The present study adds the conductivity-sensitive four-electrode path, the PCB resistance-based temperature-sensitive path, and a switching network that time multiplexes all three channels on the composite electrode. The CSF11-60 data reported here were newly collected using the soil preparations and 72 conditions of the present study rather than reused from the earlier publication.

Accordingly, this study develops a time division multiplexed three-parameter FDR sensor for in situ soil moisture measurement and establishes a joint three-parameter calibration model over temperature and salinity ranges of 5–40 °C and 0–6 g/kg, respectively. The main contributions are as follows:

1. The inherited IDE/FDR subsystem is integrated with conductivity- and temperature-sensitive paths through a composite electrode and switching network, enabling sequential acquisition of three raw responses at the same sensor location.
2. A three-input calibration workflow is established using resonant frequency, conductivity-sensitive voltage, and temperature-sensitive voltage, and input-ablation analysis is used to quantify the contribution of the added sensing channels.
3. The selected network is deployed on the STM32 platform, and the locked prototype is evaluated through repeat measurements, electrode consistency, repeatability, and short-term stability tests within the same prepared sandy loam system.

## 2. Three-Parameter FDR Soil Moisture Sensor

The sensor hardware comprises an FDR channel, a conductivity-sensitive channel, a temperature-sensitive channel, a composite electrode, an analog switching network, and an STM32F405RGT6 microcontroller (STMicroelectronics N.V., Geneva, Switzerland). Rather than operating simultaneously, the three channels are switched in a fixed sequence by the microcontroller. The same composite electrode, therefore, outputs the resonant frequency *f*, conductivity-sensitive voltage *V_EC_*, and temperature-sensitive voltage *V_T_* sequentially within one measurement cycle. Here, *V_EC_* and *V_T_* enter the subsequent calibration model directly as proxies for the conductive and temperature states; they are not interpreted as independently calibrated absolute EC and absolute temperature measurements.

### 2.1. Frequency Domain Reflectometry Channel

The FDR channel utilizes the characteristics of soil dielectric state changing with water content. When the soil surrounding the electrode acts as the dielectric, the equivalent probe capacitance *C_eq_* and an external inductance *L* form a resonant network, whose resonant frequency varies with the equivalent capacitance [32,33]. In this study, resonant frequency is retained as the raw water content-related output rather than first being converted to water content using a fixed empirical equation.

The FDR channel circuit uses an MC1648 voltage-controlled oscillator (Motorola, Schaumburg, IL, USA) to form a high-frequency oscillation network. Its output, denoted *f_osc_*, is approximately 26 MHz. The signal is amplified by a 2SC3356 high-frequency NPN transistor (NEC Corporation, Tokyo, Japan) and then divided successively by 128 using an MB504 (Fujitsu, Kawasaki, Japan) and by 256 using an SN74HC393D (Texas Instruments, Dallas, TX, USA). The microcontroller measures the interval between adjacent rising edges of the divided signal. The frequency recorded by the microcontroller and used throughout this study, denoted *f_MCU_* = *f_osc_*/(128 × 256) = *f_osc_*/32,768, is reported in kHz. Thus, *f_MCU_* rather than *f_osc_* is the water content-related frequency supplied to the calibration model and shown in the tables and figures.

### 2.2. Conductivity-Sensitive Channel

Because soil conductivity affects complex permittivity and dielectric loss, the conductivity-sensitive channel uses an AC four-electrode arrangement. Separate excitation and sensing electrodes reduce direct polarization and contact-resistance effects. The conductivity-sensitive channel consists of AC excitation, an improved Howland constant-current source, four-electrode response acquisition, differential amplification, true RMS conversion, and analog-to-digital conversion. An AD9833 direct digital synthesizer (Analog Devices, Wilmington, MA, USA) generates a 1 kHz sinusoidal signal. After DC blocking and low-pass filtering, the signal enters an improved Howland AC constant-current source built around an ADA4522-2ARZ zero-drift operational amplifier (Analog Devices, Wilmington, MA, USA). The outer electrodes inject AC current into the soil, while the inner electrodes sense the potential difference. The weak AC voltage is amplified by an AD623 instrumentation amplifier (Analog Devices, Wilmington, MA, USA), converted to a DC RMS value by an AD736 (Analog Devices, Wilmington, MA, USA), sampled by a 16-bit ADS1110 analog-to-digital converter (Texas Instruments, Dallas, TX, USA), and sent to the STM32. The ADC voltage output of this channel is denoted by *V_EC_*. Because the composite electrode combines rectangular, interdigital, and serpentine copper patterns, it does not satisfy all assumptions of the classical Wenner geometry. Moreover, its geometric factor has not been calibrated using standard solutions or independent metrology. The channel is, therefore, intended to provide a voltage proxy *V_EC_* related to salinity and conductive state, rather than an absolute measurement of soil electrical conductivity.

### 2.3. Temperature-Sensitive Channel

The temperature-sensitive channel provides temperature-state information corresponding to each FDR measurement. A serpentine PCB copper trace with a line width of 0.1 mm forms a resistive temperature-sensing element whose resistance varies with local temperature. When this element is connected to a Wheatstone bridge, the resistance change is converted to a millivolt-level differential voltage, conditioned by an AD623 instrumentation amplifier, and acquired by an ADS1110. The channel output is denoted by *V_T_*. This channel is intended to provide a temperature-related voltage proxy *V_T_*, rather than an absolute measurement of soil temperature.

### 2.4. System Architecture and Measurement Sequence

The time division multiplexing strategy addresses two direct problems. First, the three signals are acquired at the same physical sensor location with a shared composite electrode, reducing the spatial mismatch introduced by separate probes. This arrangement does not imply identical sensing volumes. The FDR response is governed by the fringing electric field, the conductivity-sensitive response by the four-electrode current path, and the temperature-sensitive response by the local serpentine copper resistance. The three channels, therefore, have spatially overlapping but channel-dependent effective support regions. Second, only one measurement circuit is active at a time, reducing interference among the high-frequency oscillator, AC excitation, and bridge measurement.

#### 2.4.1. Composite Electrode Design

**Figure 1:**
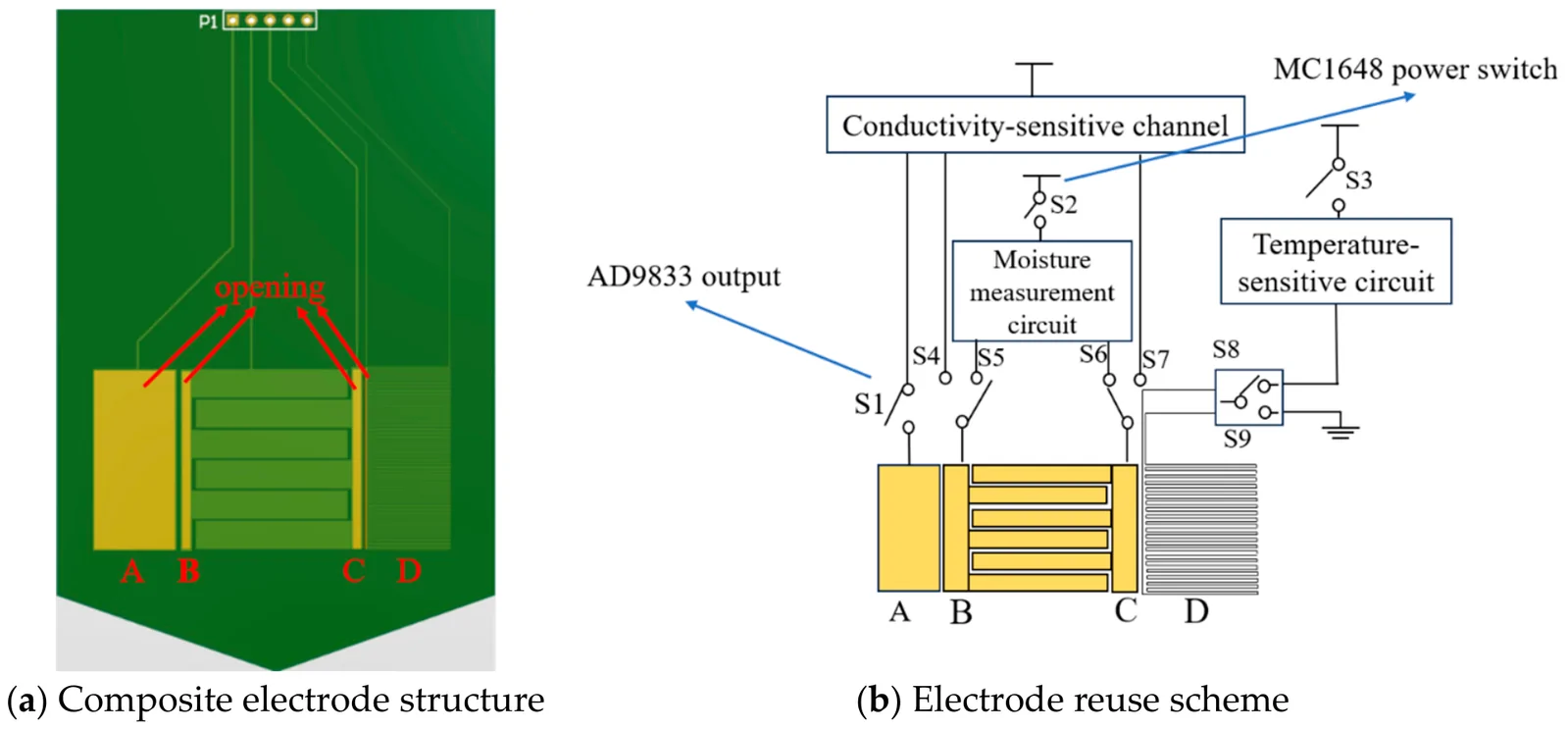
Composite electrode and electrode reuse scheme.

Figure 1a shows the four electrodes. Electrode A is rectangular; interdigital electrodes B and C form the FDR capacitance; and the serpentine PCB copper trace D acts as the temperature-sensitive resistor. The B-C geometry produces a fringing field within a limited area and follows the previously reported structure [6,7]. Its gap is 0.3 mm, with a finger width of 3.93 mm, a finger length of 21.492 mm, and a finger count of six.

The B-C layout and the MC1648-based FDR signal-conditioning chain follow the authors’ earlier electrode and FDR studies [6,7]. In the present sensor, they are retained as the moisture-responsive subsystem and integrated with the newly added A-D electrode functions, conductivity- and temperature-sensitive circuits, and switching network described below. The contribution of the composite electrode is, therefore, this functional integration and sequential three-channel use, not a new claim for the underlying IDE geometry or FDR principle.

Figure 1b shows electrode reuse. In conductivity-sensitive mode, A and D inject current while B and C sense potential. In FDR mode, B and C connect to the MC1648 network; in temperature-sensitive mode, D connects to the Wheatstone bridge. Thus, the same electrode set serves three functions sequentially at one location.

The PCB layout separates the two functions assigned to electrode D. The soil-contacting current injection portion is selectively exposed through an opening in the top solder layer (as shown in Figure 1a), whereas the adjacent serpentine resistance measurement trace remains covered by the green top solder mask. No additional spray coating, potting compound, or separate waterproof treatment was applied. Protection of the temperature-sensitive copper trace, therefore, relies on selective solder mask coverage, which reduces direct ionic contact and conductive shunting but does not establish complete independence from the surrounding soil state.

#### 2.4.2. Measurement Sequence

**Figure 2:**
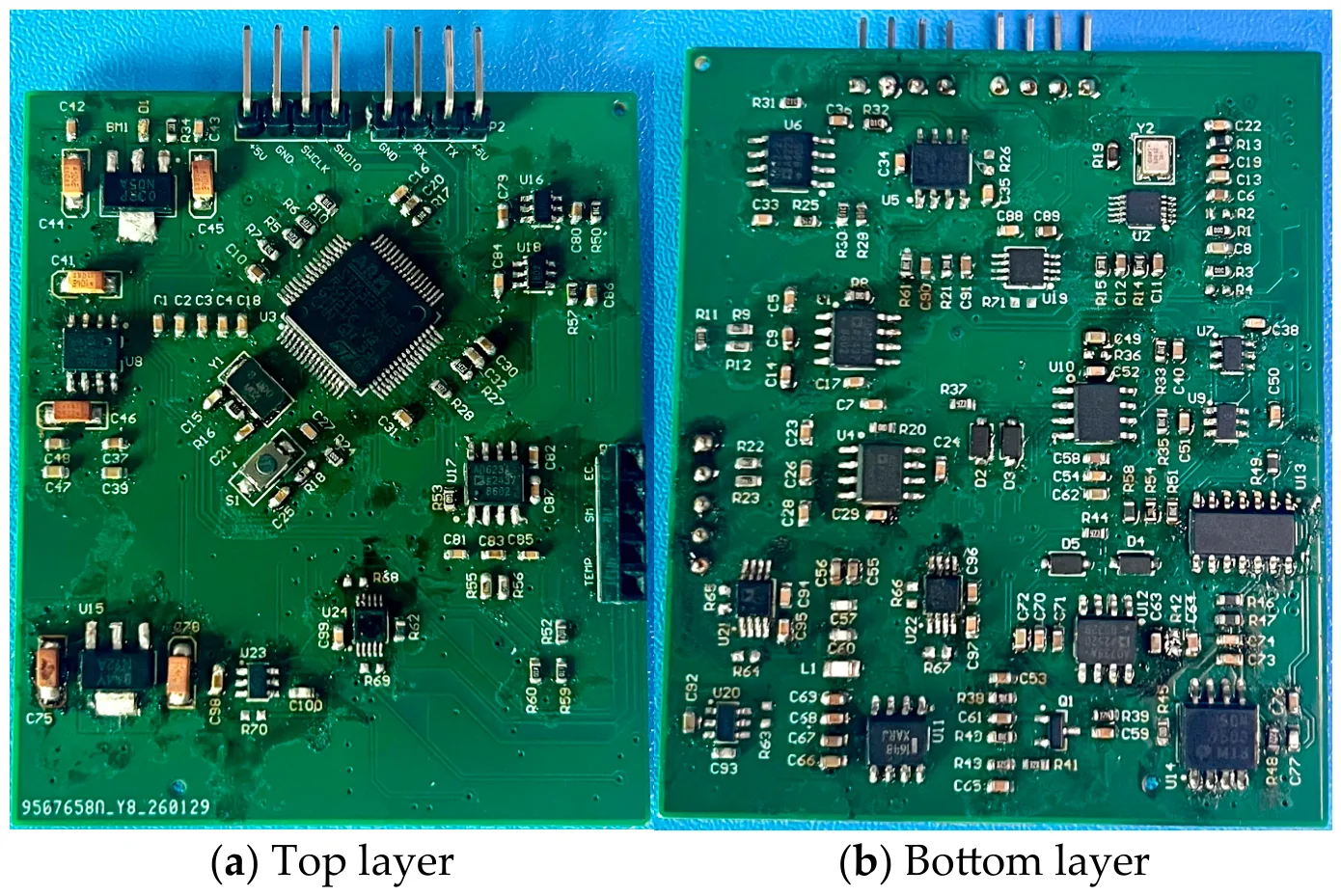
Hardware prototype of the multiparameter FDR sensor.

Figure 1b and Table 1 summarize channel switching. Conductivity acquisition closes S1, S4, S7, and S9; FDR closes S2, S5, and S6; and temperature acquisition closes S3 and S8. S1 (ADG1419; Analog Devices, Wilmington, MA, USA) switches the AD9833 excitation, while S2 and S3 (TPS22918; Texas Instruments, Dallas, TX, USA) control oscillator and bridge power. S4–S7 (ADG619; Analog Devices, Wilmington, MA, USA) route B and C, and S8–S9 (ADG884; Analog Devices, Wilmington, MA, USA) switch D between the conductivity and temperature paths. Figure 2 shows the prototype.

The firmware polls the conductivity-sensitive, FDR, and temperature-sensitive modes in that order. For the conductivity-sensitive mode, the firmware switches the electrode path, connects the AD9833 excitation, and waits 100 ms before voltage acquisition. For the FDR mode, it switches the electrode path, enables the MC1648 oscillator, and waits 100 ms before frequency measurement. For the temperature-sensitive mode, it connects the temperature-sensitive electrode and waits 200 ms, then powers the temperature bridge and waits another 100 ms before voltage acquisition. Together with the preceding circuit-disable and electrode-switching delays, the total programmed delay before the first acquisition was 600 ms in each mode. The conductivity-sensitive and temperature-sensitive voltages were each averaged from 10 ADS1110 readings, with a 100 ms interval after each read. The FDR output was averaged from 10 adjacent rising-edge period measurements, with a 50 ms interval between measurements. No initial samples were explicitly discarded. The AD736 RMS converter used CAV = 0.1 µF. These firmware-defined pre-acquisition delays were applied consistently throughout calibration and validation. The three channel outputs are assembled into one input vector for the calibration model. Time division multiplexing, therefore, reduces simultaneous interference among the three circuits, but it does not by itself eliminate physical cross-sensitivity of their raw responses; any remaining coupled dependence is retained in the three-input calibration model.

## 3. Development and Selection of the Three-Parameter Calibration Model

### 3.1. Soil Samples and Multidimensional Experimental Design

**Figure 3:**
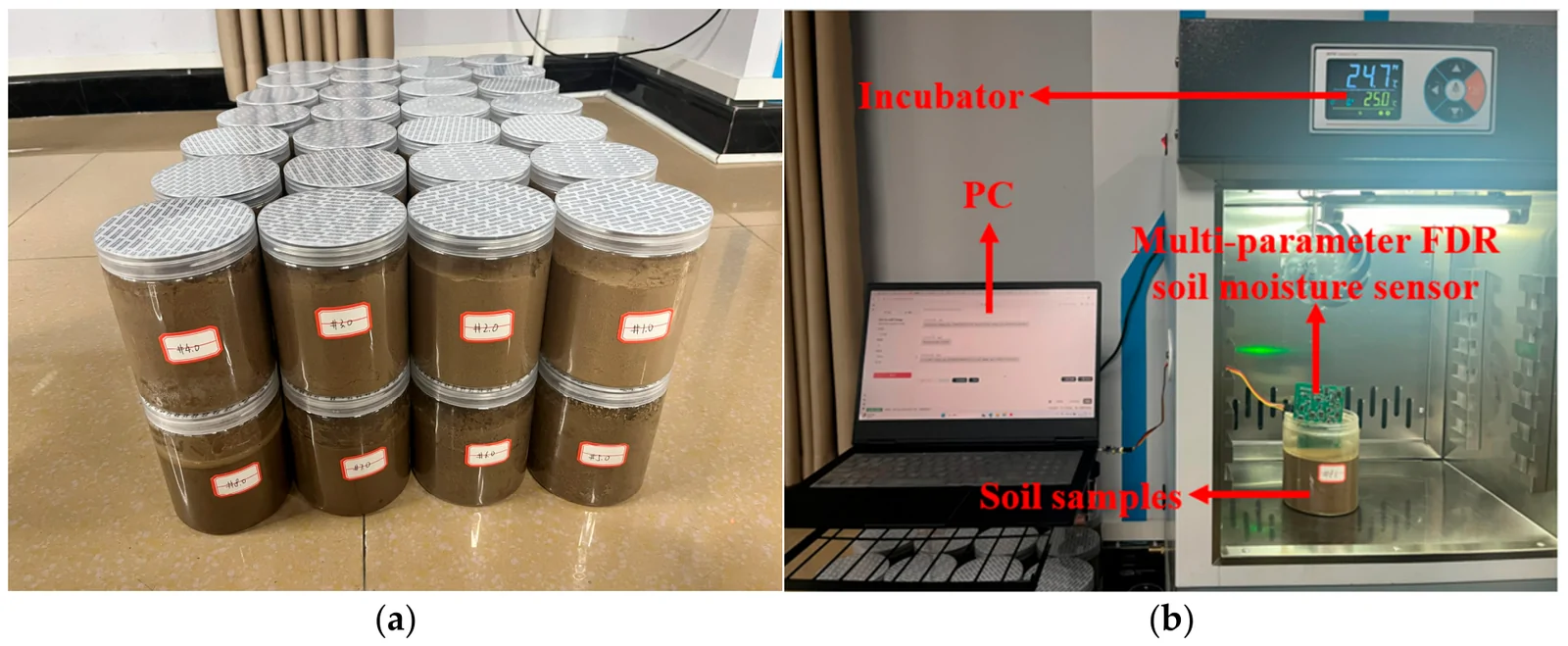
Soil samples and data acquisition platform. (**a**) Physical soil samples with different water contents and salinities; (**b**) sensor acquisition platform in the incubator.

Table 2 summarizes the experimental design of the three-parameter calibration dataset. The sandy loam soil was collected in Qingjiangpu District, Huai’an, Jiangsu Province, China. Large particles and roots were removed using a 2 mm sieve, and the soil was oven-dried with forced air at 105 °C for 24 h. Each portion of 1 kg dry soil was then placed in a cylindrical container that was 100 mm in diameter and 120 mm high. Eight water content levels were set at intervals of approximately 5 percentage points. Salinity was adjusted by adding NaCl to deionized water at seven levels: 0, 1, 2, 3, 4, 5, and 6 g/kg. Each water content salinity combination was mixed uniformly, sealed, and equilibrated for 48 h. The 56 water content salinity samples and one blank control containing neither added salt nor water produced 57 physical soil samples (Figure 3a).

To prepare the water content and salinity treatments, the required water addition for each nominal water content level was obtained from a preliminary quadratic fit between added water and oven-drying volumetric water content. For each 1 kg dry soil portion, 0–6 g of NaCl was dissolved in the corresponding amount of deionized water. The solution was applied with a spray bottle, after which the soil was mixed thoroughly and returned to the cylindrical container. The soil was not packed in separate layers; the container was tapped to settle the mixture and then covered with a moisture-proof lid for 48 h. The dry bulk density of the prepared sandy loam soil was 1.522 g/cm^3^. During measurement, the sensor was inserted near the center of the container until the active electrode region was completely covered by soil.

Reference volumetric water content was determined by oven drying. A cutting ring was used to collect soil; the masses of the wet soil and container were recorded, and volumetric water content was calculated after drying at 105 °C for 24 h. Reference soil electrical conductivity was measured using the 1:5 soil–water extract method (EC1:5). After 20 g of air-dried soil was mixed with 100 mL of deionized water, stirred, and allowed to stand, the supernatant EC1:5 was measured using a temperature-compensated DDS-307 conductivity meter (Shanghai INESA Scientific Instrument Co., Ltd., Shanghai, China). EC1:5 was used only to describe sample salinity and analyze response curves; the actual model input remained the sensor’s conductivity-sensitive voltage *V_EC_*. After each water content and salinity sample had been prepared and homogenized, a subsample was taken from that same prepared soil sample before the temperature experiment. Its volumetric water content was determined by oven drying at 105 °C for 24 h. This initial value was used as the reference for the corresponding physical sample at all temperature points; a separately prepared parallel sample was not used.

Each soil sample was placed sequentially in an incubator at eight temperature levels from 5 to 40 °C in 5 °C increments. After the soil temperature reached the set point and stabilized, the sensor was inserted, and 10 complete three-parameter readings were acquired for each physical sample at each temperature (Figure 3b). The 56 water content salinity samples across eight temperature levels produced 448 sample conditions, and the blank control produced another eight conditions. In total, the dataset contained 456 combined conditions and 4560 records. The same physical sample was tested in ascending order from 5 to 40 °C. Because the time required for thermal equilibration depended on temperature, water content, and salinity, a single fixed equilibration interval was not used; acquisition began only after the monitored soil temperature reached and remained at the set point. The moisture-proof lid remained closed during equilibration and was removed only for sensor insertion and data acquisition, for approximately 5 min at each temperature. The soil was not remixed between temperature points.

### 3.2. Response Characterization: Effects of Temperature and Salinity on Resonant Frequency

This section examines the raw output *f* of the FDR water content channel. Comparing resonant frequencies at different EC1:5 values and set temperatures under the same water content determines whether temperature and salinity interference is sufficient to destabilize single-frequency water content mapping. The following analysis uses the mean of 10 readings for each condition; the blank control is excluded from the response-pattern statistics.

**Figure 4:**
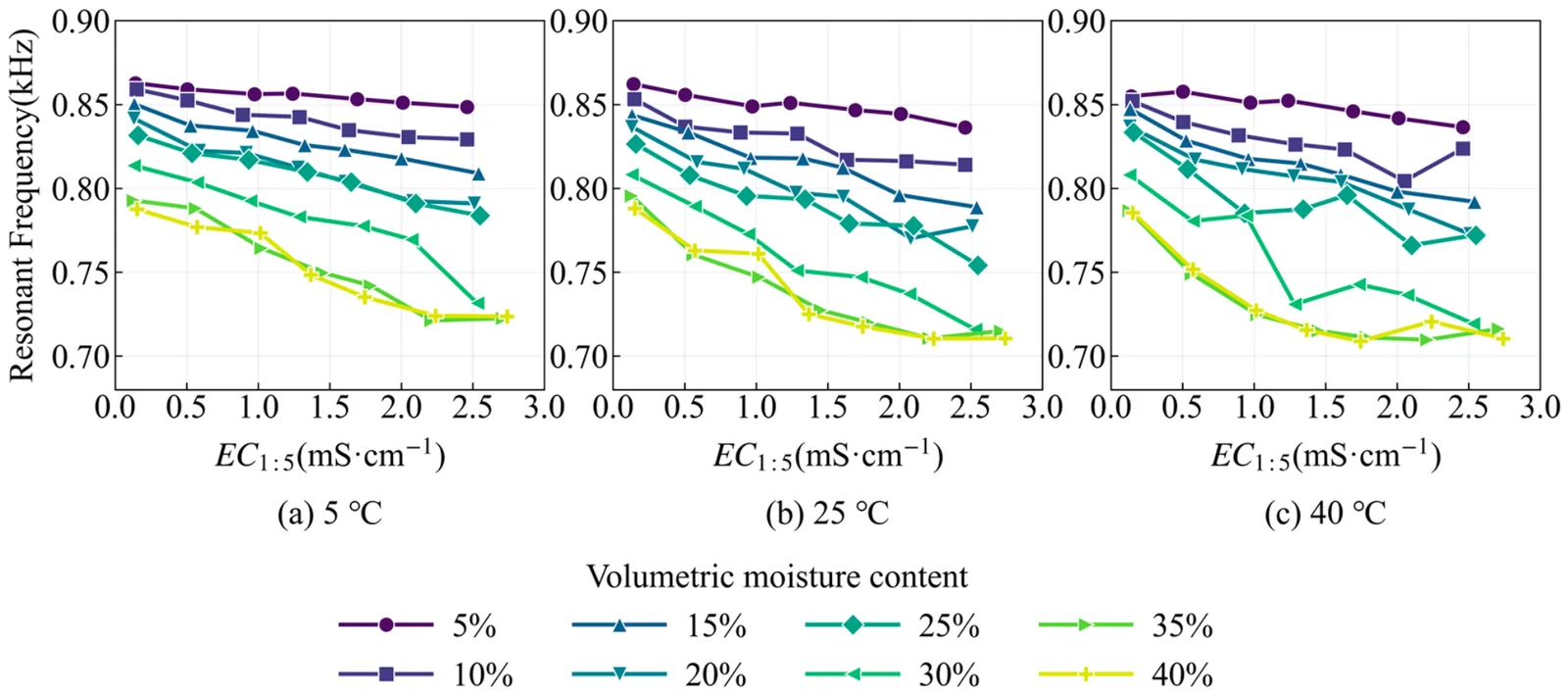
Effect of EC1:5 on resonant frequency at different temperatures.

Figure 4 shows resonant frequency versus EC1:5 at 5, 25, and 40 °C. Frequency generally decreased as EC1:5 increased, but the magnitude depended on water content. Absolute linear fit slopes ranged from 5.769 to 30.996, 9.708 to 36.993, and 8.685 to 35.738 Hz/(mS·cm^−1^) at 5, 25, and 40 °C, respectively. Thus, salinity does not produce a fixed, water content-independent offset.

**Figure 5:**
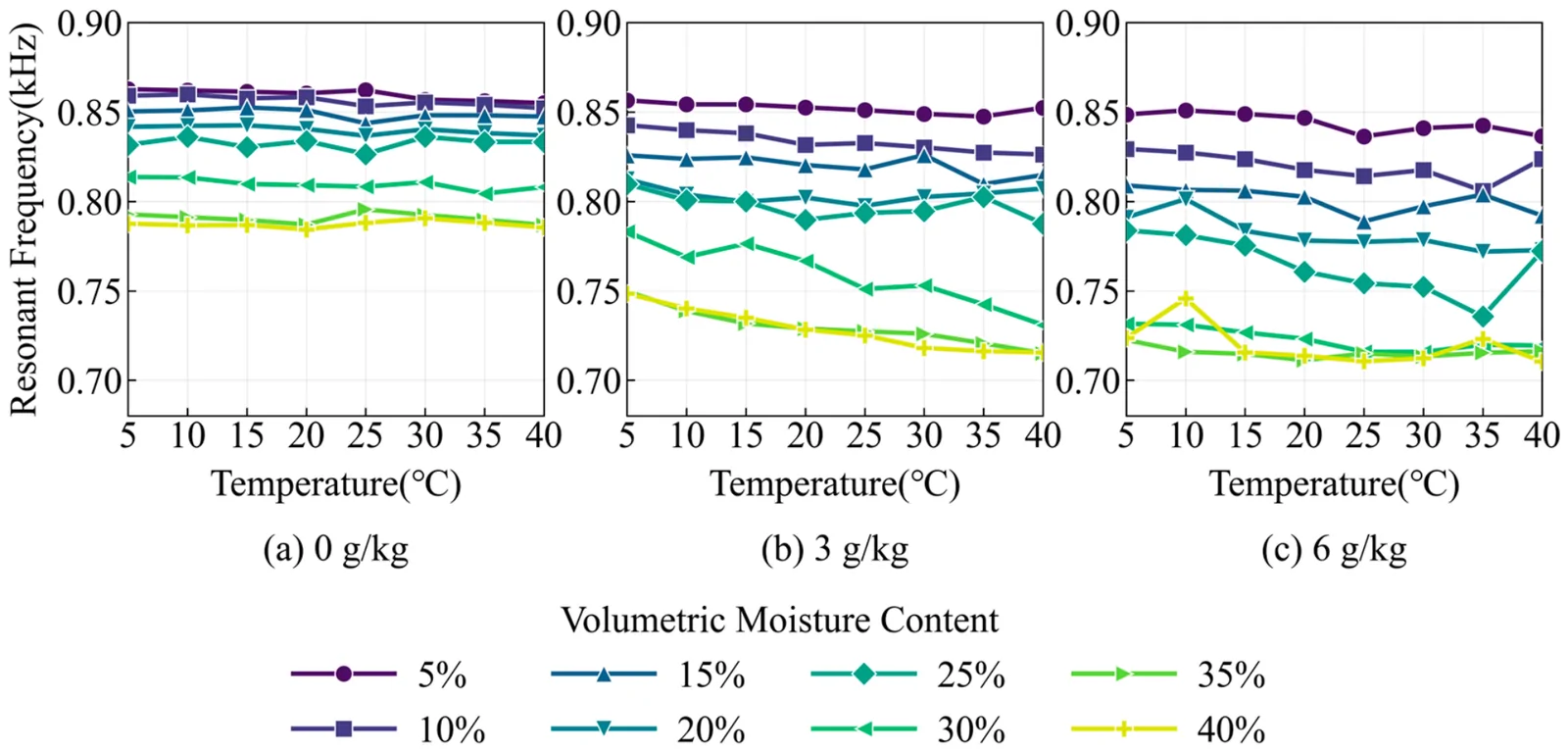
Effect of temperature on resonant frequency at different salinities.

Figure 5 shows resonant frequency versus temperature at salinities of 0, 3, and 6 g/kg. Temperature sensitivity ranged from −0.227 to 0.019, −1.388 to −0.072, and −0.917 to −0.113 Hz/°C, respectively. Local nonmonotonic fluctuations indicate that polarization, ion transport, probe materials, and circuit drift can act in different directions; one global linear temperature coefficient, therefore, cannot cover all salinity and water content states.

**Figure 6:**
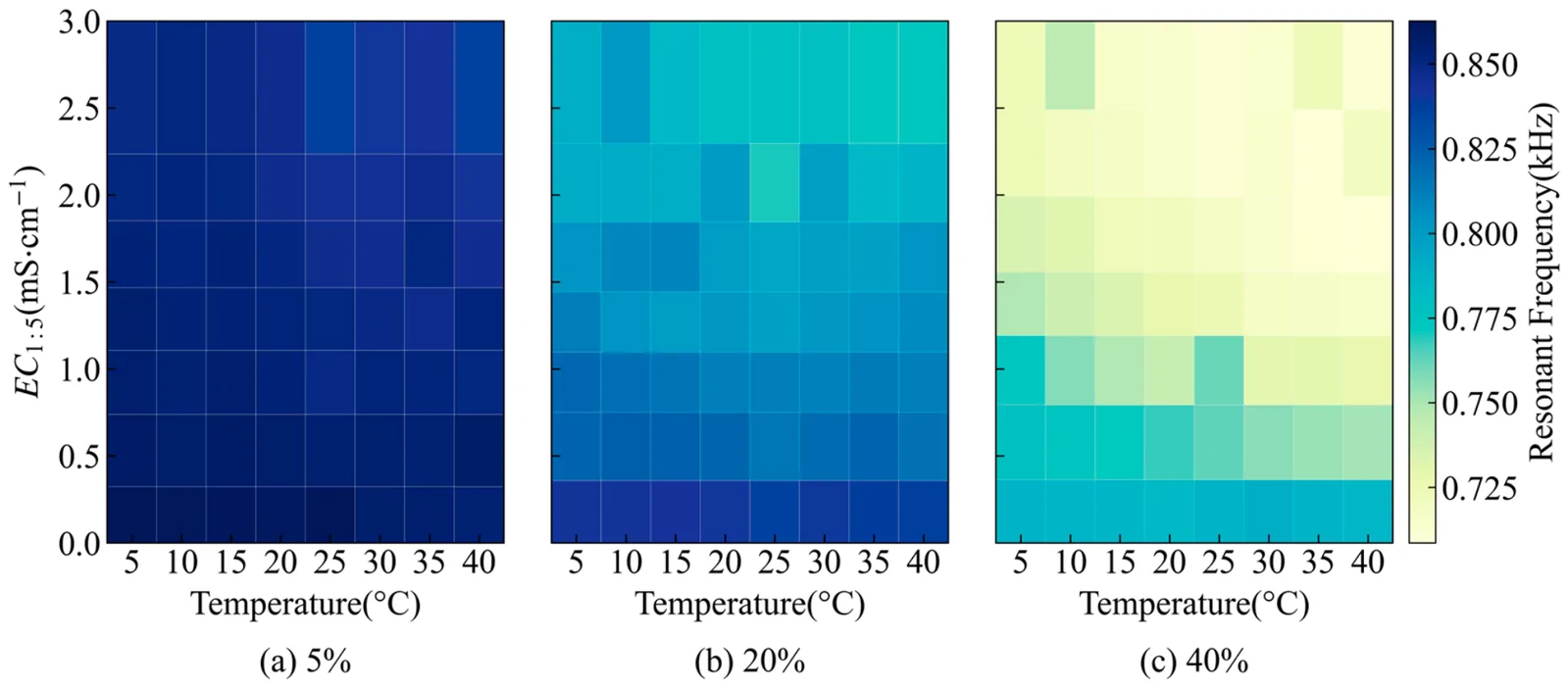
Joint response of resonant frequency to temperature and EC1:5.

To examine the overall magnitude of the joint temperature and salinity response, measured-grid heatmaps were plotted for three representative volumetric water content levels of 5%, 20%, and 40% (Figure 6a–c). Each color block represents only the mean of 10 readings under the corresponding condition; no response surface fitting or interpolation was performed. The resonant frequency ranges at the three water content levels were 26.437, 72.323, and 81.922 Hz, respectively (Table 3), with substantially greater variation at medium and high water contents than at low water content. These results show that resonant frequency is affected jointly by temperature and salinity and that the magnitude of interference depends on water content state, providing direct experimental support for including *f*, *V_EC_*, and *V_T_* simultaneously.

To examine the conductivity channel response near the upper salinity boundary, the 10 readings for each water content–salinity–temperature condition were averaged. For each water content–temperature combination, the absolute *V_EC_*–EC1:5 slope over 3–6 g/kg was compared with that over 0–3 g/kg. The high-salinity slope was smaller in 63 of the 64 combinations, indicating that *V_EC_* generally changed less as EC1:5 continued to increase at high salinity.

### 3.3. Definition of the Three-Parameter Input and Output

The input to the three-parameter calibration model consists of the resonant frequency *f*, conductivity-sensitive voltage *V_EC_*, and temperature-sensitive voltage *V_T_* obtained within the same measurement cycle and is written as $x={\left[f,V_{\mathrm{EC}},V_{T}\right]}^{T}$. The target *y* is the reference volumetric water content determined by oven drying. The resonant frequency *f* reflects the dielectric and water content state, *V_EC_* provides information on salinity and conductive state, and *V_T_* provides temperature state information. For brevity, the symbol *f* in the subsequent equations, tables, and figures refers to *f_MCU_*, not to the approximately 26 MHz oscillator output *f_OSC_*. Rather than first estimating electrical conductivity and temperature and then applying successive corrections, the model directly learns the nonlinear mapping from the three raw outputs to volumetric water content.

The 10 raw readings from each condition were retained without prior averaging, so all 4560 records participated in training and outer evaluation. For each training partition, the three inputs and the target were standardized using the mean *μ* and standard deviation *σ* calculated from that partition alone. Validation folds were never used to calculate the standardization parameters, which prevented test fold information from entering training through preprocessing. Model outputs were then inverse-transformed to volumetric water content percentages using the target mean and standard deviation of the corresponding training partition. Standardization was performed as follows:

(1) $$x_{j}^{\prime }=\frac{x_{j}-\mu_{j,\mathrm{train}}}{\sigma_{j,\mathrm{train}}}$$

### 3.4. Evaluation Protocol and Control of Information Leakage

Each physical sample contributed 80 records (eight temperatures and 10 measurements per temperature) that shared preparation and contact conditions. The sample identifier #x.y was, therefore, used as the group and water content level as the stratification label. StratifiedGroupKFold generated five outer folds, and hyperparameters were selected through another five-fold grouped split within each outer training set, preventing records from one sample from entering both training and test data.

All models used identical outer sample groups. Metrics pooled the 4560 outer test predictions, and the random seed was 42. Table 4 summarizes the nested validation and preprocessing.

The evaluation metrics were the coefficient of determination *R*^2^, root mean square error (RMSE), mean absolute error (MAE), systematic bias (Bias), the 95th percentile of absolute error P95(|e|), and maximum absolute error (MaxAE). Except for *R*^2^, all metrics are expressed in percentage points (pp) of volumetric water content. Unless otherwise stated, *R*^2^ and the error metrics are reported to three decimal places. In the repeated-seed analysis, *R*^2^ is reported to four decimal places because the between-seed standard deviation occurs at the fourth decimal place; percentage reductions are reported to one decimal place.

### 3.5. Performance Comparison and Selection of Five Calibration Models

Five models were compared: BPNN, random forest (RF), extreme gradient boosting (XGBoost), polynomial regression, and support vector regression (SVR). BPNN used a fully connected 3-128-64-1 architecture with ReLU hidden layers, a linear output, mean squared error loss, and Adam optimization.

All comparison models used the same three inputs and outer folds. Polynomial regression included linear terms, quadratic terms, pairwise interactions, and a three-way interaction. The final RF configuration used 150 trees, a maximum depth of 24, and a maximum feature fraction of 0.8. XGBoost used 600 trees, a maximum depth of 4, a learning rate of 0.1, and a subsampling fraction of 0.8. SVR used a radial basis function (RBF) kernel with C = 500, γ = 10, and ε = 0.05. Candidate hyperparameters were compared only within the inner grouped folds of the corresponding outer training set.

Table 5 presents the outer grouped five-fold validation performance of the five calibration models. BPNN achieved the highest *R*^2^ (0.911) and the lowest RMSE (3.500 percentage points), MAE (2.761 percentage points), and P95(|e|) (6.575 percentage points), giving the best overall performance among the baseline models. BPNN did not lead on every statistic: polynomial regression had a smaller absolute bias, and XGBoost had the lowest MaxAE. Its advantage was, therefore, concentrated in overall fit, average error, and the 95th percentile error level.

**Figure 7:**
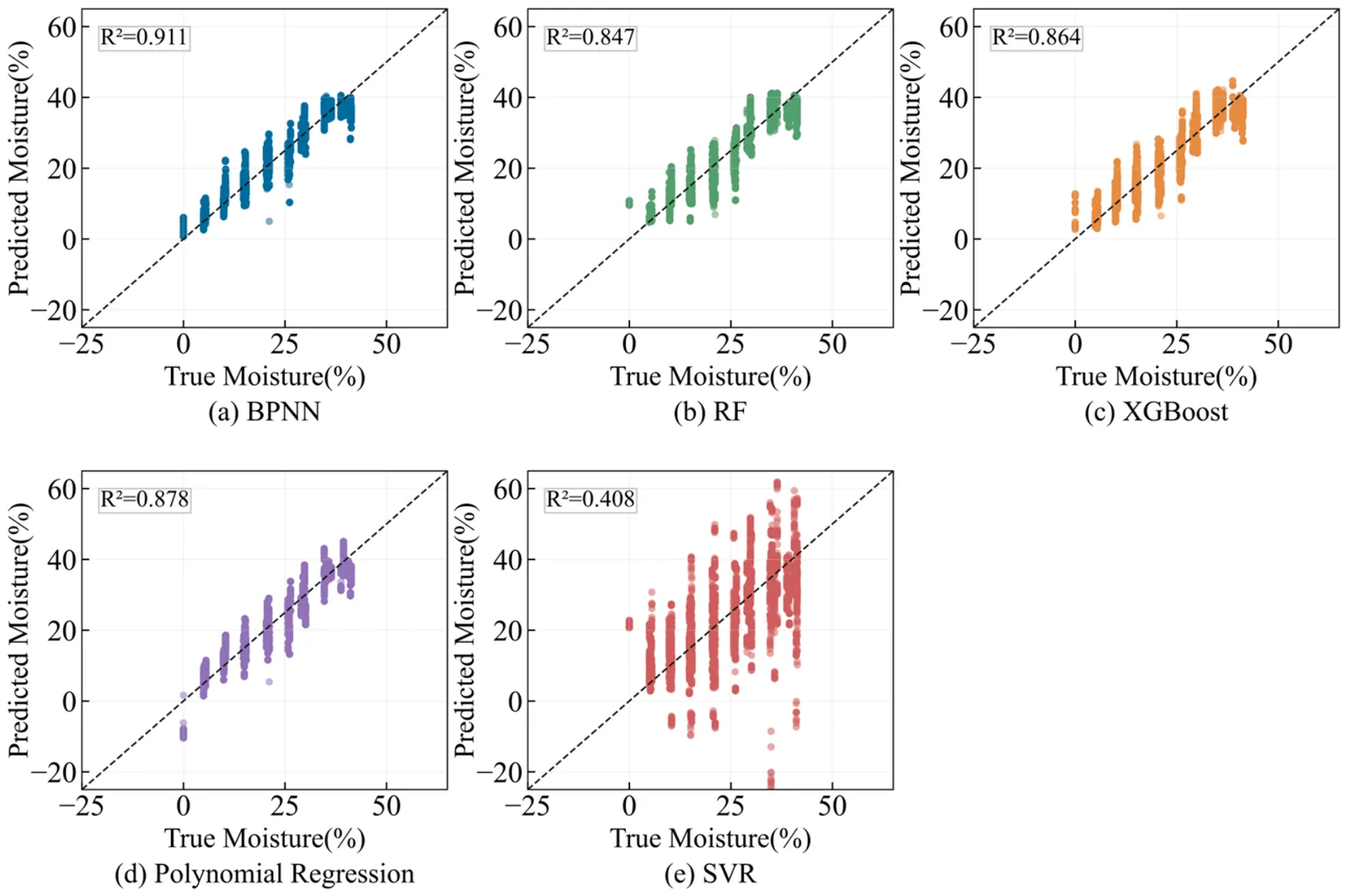
Relationship between predicted and true values under outer validation for the five calibration models.

Figure 7 shows that BPNN predictions were generally closest to the 1:1 line, although dispersion remained at medium and high water contents. SVR showed the widest scatter, consistent with its RMSE of 9.045 and MaxAE of 59.405 percentage points.

These metrics and embedded implementation requirements motivated the selection of BPNN as the base network for initialization analysis.

### 3.6. Contribution of Individual Input Parameters

To isolate the contribution of each sensing channel from the choice of regression algorithm, an input ablation analysis was performed using the BPNN selected in Section 3.5. Four input configurations were compared: resonant frequency alone (*f*), *f* with conductivity-sensitive voltage (*f* + *V_EC_*), *f* with temperature-sensitive voltage (*f* + *V_T_*), and all three inputs (*f* + *V_T_* + *V_EC_*). All configurations used the same outer folds, fold-wise standardization, nested grouped five-fold validation procedure, hyperparameter search space, and random seed. The network architecture and training procedure were unchanged except for the input-layer dimension.

Table 6 shows that *V_EC_* was the main source of improvement beyond resonant frequency. Relative to the frequency-only model, adding *V_EC_* increased *R*^2^ from 0.763 to 0.906 and reduced RMSE from 5.722 to 3.598 percentage points, corresponding to a 37.1% reduction. In contrast, adding *V_T_* alone increased *R*^2^ to 0.779 and reduced RMSE to 5.526 percentage points, a reduction of 3.4%. The complete three-input model achieved the best pooled *R*^2^, RMSE, MAE, and P95(|e|), with values of 0.911, 3.500, 2.761, and 6.575 percentage points, respectively.

Compared with *f* + *V_EC_*, adding *V_T_* further reduced RMSE by 0.098 percentage points, MAE by 0.115 percentage points, and P95(|e|) by 0.231 percentage points. This incremental improvement was modest and did not extend to bias or MaxAE: *f* + *V_EC_* produced the smallest absolute bias, whereas the frequency-only model had the lowest MaxAE. These results support the three-input configuration for improving overall fit, average error, and the 95th percentile error level, while indicating that most of the additional predictive information was provided by the conductivity-sensitive channel.

#### Cross-Sensitivity of the Temperature-Sensitive Voltage Proxy

The cross-sensitivity of the temperature-sensitive voltage was evaluated using all 4560 raw readings (as shown in Table 7). The 10 consecutive readings from each of the 456 temperature–soil sample conditions were averaged so that the physical condition, rather than each technical repeat, was the analysis unit. A multivariable linear model related *V_T_* to temperature, reference volumetric water content, and NaCl salinity. Coefficient uncertainty was estimated by 4000 bootstrap resamples at the physical soil sample level. Separate regressions at each temperature were also used to examine moisture and salinity effects while temperature was fixed.

Across the full dataset, temperature was the dominant predictor of *V_T_*. The estimated temperature coefficient was 0.004472 V/°C (95% CI: 0.004385–0.004560 V/°C), the standardized coefficient was 0.987, and removing temperature reduced *R*^2^ by 0.974. In comparison, the average coefficients for volumetric water content and salinity were −0.000566 V per 10 percentage points (95% CI: −0.001752 to 0.000413 V) and 0.000039 V/(g/kg) (95% CI: −0.000584 to 0.000659 V), respectively. Adding all pairwise interactions increased *R*^2^ by only 0.0010.

The fixed-temperature analysis nevertheless showed that *V_T_* was not completely independent of the soil state. The water content coefficient ranged from −0.005795 to 0.001319 V per 10 percentage points across the eight temperatures. Its bootstrap interval excluded zero at 5 °C (−0.008216 to −0.003278 V per 10 percentage points), whereas the intervals at the other temperatures included zero. The salinity coefficient ranged from −0.000904 to 0.000823 V/(g/kg), and all temperature-specific bootstrap intervals included zero. Accordingly, *V_T_* is described as a temperature-sensitive voltage proxy rather than an independent temperature measurement. The insulation and multiplexing reduce direct interference, while the joint model uses the remaining correlated responses from all three channels.

### 3.7. Repeated-Seed Assessment of WOA Initialization

WOA was evaluated only as an offline initializer for the BPNN weights and biases [34]. The input variables, 3-128-64-1 architecture, 8833 trainable parameters, Adam optimizer, and embedded forward-inference procedure were otherwise unchanged. The WOA configuration used a population of 12, 35 iterations, a search radius of 0.25, and a spiral parameter b of 1.5. Thus, WOA added offline optimization but did not change the memory requirement or inference operations of the deployed network.

The original single nested grouped cross-validation run used random seed 42 and selected hyperparameters and training duration within each outer fold. In that run, RMSE decreased only from 3.500 percentage points for BPNN to 3.493 percentage points for WOA-BPNN, while P95(|e|) and MaxAE increased. To assess whether this 0.007 percentage point difference was stable, both models were subsequently evaluated with the paired seeds 2, 12, 22, 32, 42, 52, 62, 72, 82, and 92. The repeated-seed analysis used identical predefined outer folds, fold-wise standardization, the same architecture, an Adam learning rate of 0.01, a weight decay coefficient of 0.0001, and 200 Adam epochs per outer fold. A fixed configuration was used in this analysis to isolate variability from random initialization; it, therefore, differs from the earlier fold-specific nested model selection procedure.

Across the 10 seeds, BPNN achieved *R*^2^ = 0.9099 ± 0.0016 and RMSE = 3.528 ± 0.031 percentage points, whereas WOA-BPNN achieved *R*^2^ = 0.9041 ± 0.0033 and RMSE = 3.639 ± 0.063 percentage points. The paired RMSE difference (WOA-BPNN minus BPNN) was 0.112 percentage points (95% CI: 0.049–0.175), and WOA-BPNN had a lower RMSE in only one of the 10 paired seeds. Its mean MAE, P95(|e|), MaxAE, and absolute bias were also higher (Table 8). The measured total offline training time across the five outer folds was 2.211 ± 1.230 s per seed for BPNN and 5.497 ± 1.124 s per seed for WOA-BPNN on the same computer; WOA also required 2160 candidate evaluations per seed across the five folds. The timing values are machine-specific, but they show added offline cost without improved mean accuracy. BPNN was, therefore, selected as the preferred default calibration model, and WOA initialization was retained only as a secondary robustness analysis.

The earlier single-run result and the repeated-seed result estimate different procedures and are not expected to coincide numerically. In particular, the earlier result used fold-specific inner model selection and training duration selection, whereas the repeated-seed analysis fixed the configuration and training budget. The repeated-seed analysis was completed after the prototype measurements. The Section 4 measurements, therefore, remain validation of the specific frozen WOA-initialized BPNN instance that had already been retrained on all development records and deployed on the STM32. They are not used as evidence that WOA is superior, and they are not relabeled as results from a newly deployed standard BPNN.

## 4. Sensor Performance Validation

After the model was fixed, repeat measurements and functional tests were conducted using the same prepared sandy loam system. The repeat measurements were performed only after model training was completed, the network parameters were fixed, and the model was deployed on the STM32 platform. This section, therefore, refers to the experiment as repeat measurement validation within the same prepared sandy loam system after model deployment and does not combine it with the test set results in Section 3. In addition to overall accuracy, the results are reported by temperature and salinity, along with between-electrode agreement, within-electrode repeatability, and short-term stability, to identify error sources and operating boundaries beyond the mean metrics. The deployed instance was the frozen WOA-initialized BPNN described in Section 3.7; the measurements validate that specific prototype and do not establish an advantage of WOA over standard BPNN.

### 4.1. Sensor Repeat Measurements

The repeat measurement experiment comprised eight water content levels, three salinity levels (0, 3, and 6 g/kg), and three temperature levels (5, 25, and 40 °C), yielding 72 conditions. Each condition was represented by the mean of 10 readings.

Table 9 reports the overall and grouped performance of the sensor repeat measurements. Across all 72 conditions, *R*^2^ was 0.968, RMSE was 2.086 percentage points, MAE was 1.582 percentage points, and bias was 0.075 percentage points. When grouped by temperature, the RMSE values at 5, 25, and 40 °C were 2.349, 1.966, and 1.918 percentage points, respectively, with no monotonic deterioration as temperature increased. When grouped by salinity, the RMSE values at 0, 3, and 6 g/kg were 1.374, 1.980, and 2.692 percentage points, respectively. At 6 g/kg, *R*^2^ decreased to 0.947 and bias increased to 0.686 percentage points, indicating that high salinity remained the main source of error in the current system.

**Figure 8:**
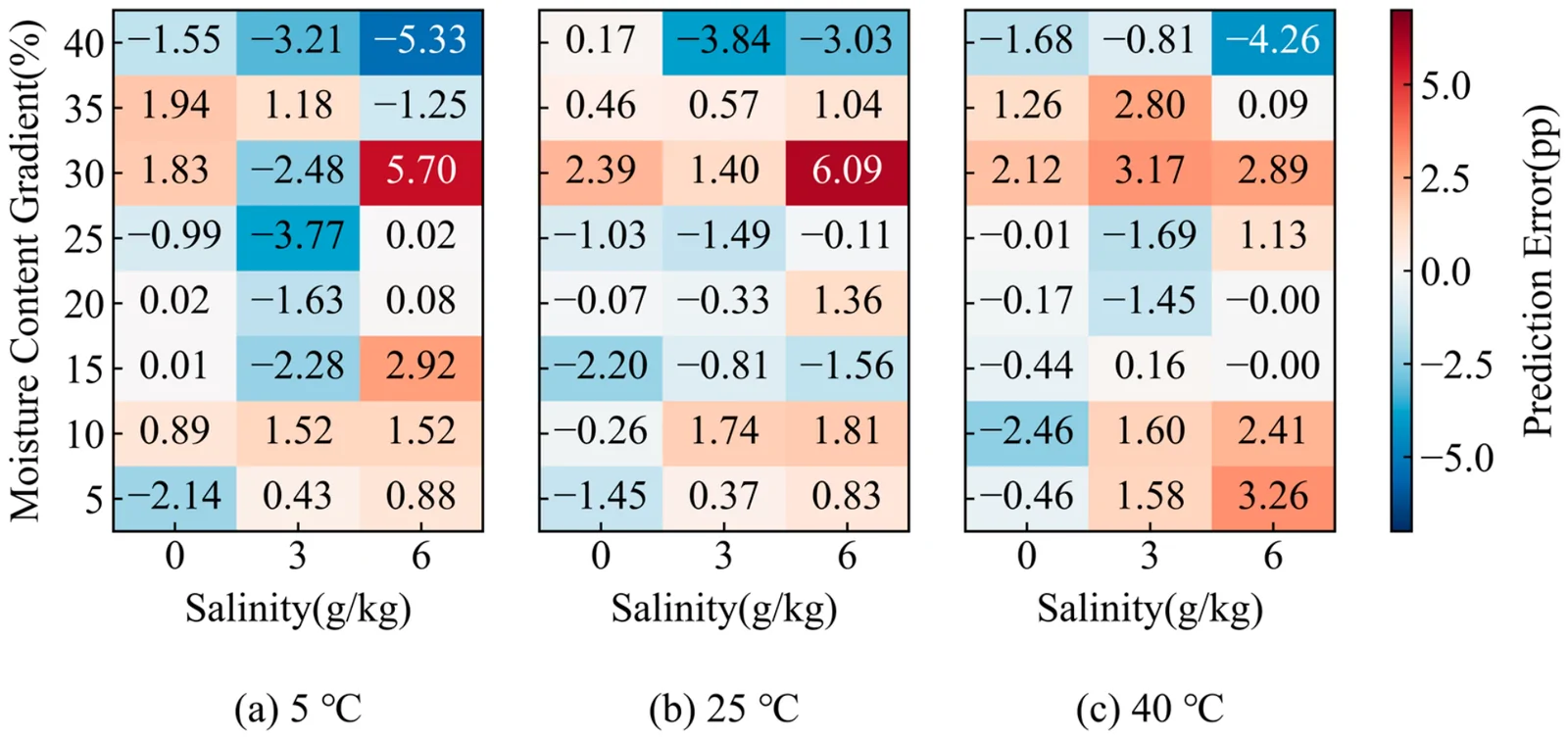
Distribution of sensor repeat measurement errors across temperature, salinity, and water content conditions within the same prepared sandy loam system. “(pp)” represents percentage points of volumetric water content.

Figure 8 maps repeat measurement errors across water content, temperature, and salinity conditions. Errors of 5.70 and 6.09 percentage points occurred at 5 and 25 °C, respectively, at 6 g/kg and the 30% water content level; at 40 °C, 6 g/kg, and 40% water content, the error was −4.26 percentage points. Thus, error direction depended on water content as well as temperature and salinity.

The data organization and evaluation objectives in Section 3 and this section differ, so their performance metrics should not be compared directly. Section 3 used outer validation grouped by physical soil sample. Each outer training set contained approximately 3648 records, and predictions were generated for soil samples excluded from that training set, primarily evaluating between-sample generalization to unseen physical samples. In Section 4, the parameter-locked deployment model was retrained on all 4560 development readings and used to remeasure 72 conditions within the same prepared sandy loam system. Each evaluation unit was the mean of 10 readings for a condition, and the temperature range was limited to 5, 25, and 40 °C. The higher *R*^2^ and lower RMSE in this section are, therefore, associated with full-data training, within-condition averaging, and a narrower validation range. They indicate repeat measurement performance for the locked deployment model under the selected conditions but do not establish generalization across other soil textures, field sites, independently prepared sample batches, or seasons.

### 4.2. Between-Electrode Agreement

Agreement among three electrodes was assessed under 27 combinations of three water content, three salinity, and three temperature levels, using means of 10 readings. Table 10 gives ICC(A,1) = 0.981 and a mean between-electrode standard deviation of 1.627 percentage points. Condition #1.6 at 40 °C produced the largest standard deviation and range, 4.682 and 9.033 percentage points, respectively.

All electrodes used the same fixed WOA-BPNN, input standardization, weights, biases, and output inverse standardization; none received electrode-specific retraining, rescaling, or offset correction.

**Figure 9:**
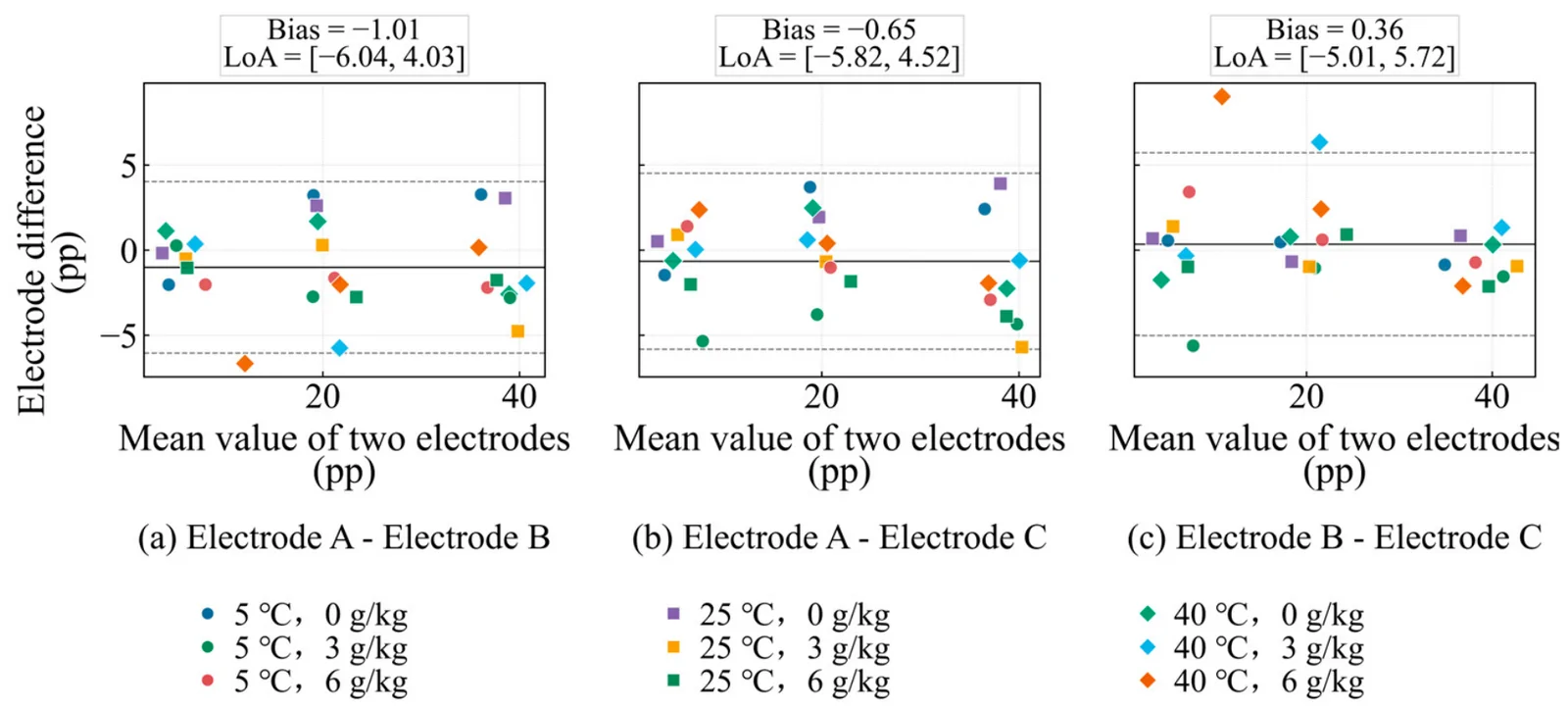
Pairwise Bland–Altman agreement analysis of the three composite electrodes. “(pp)” represents the percentage points of volumetric water content.

A high ICC does not imply that the electrodes are unconditionally interchangeable, so the Bland–Altman analysis in Figure 9 must also be considered. The mean differences for electrode pairs A-B, A-C, and B-C were −1.007, −0.651, and 0.355 percentage points, respectively, and the corresponding 95% limits of agreement were [−6.044, 4.031], [−5.821, 4.518], and [−5.013, 5.723] percentage points. Limits of agreement of approximately ±5–6 percentage points indicate that manufacturing variation, insertion state, and local soil contact can still produce between-electrode differences. Electrode-level checks or field calibration are, therefore, advisable before deployment.

### 4.3. Within-Electrode Repeatability

Repeatability was evaluated from 10 reinsertion predictions under 27 conditions. Table 11 reports a pooled standard deviation of 0.296 percentage points, a mean condition-specific standard deviation of 0.227 percentage points, and a mean coefficient of variation of 2.541%. The condition-specific standard deviation ranged from 0.009 percentage points (#8.0, 25 °C) to 0.736 percentage points (#8.6, 25 °C), with a maximum repeatability limit of 2.061 percentage points.

**Figure 10:**
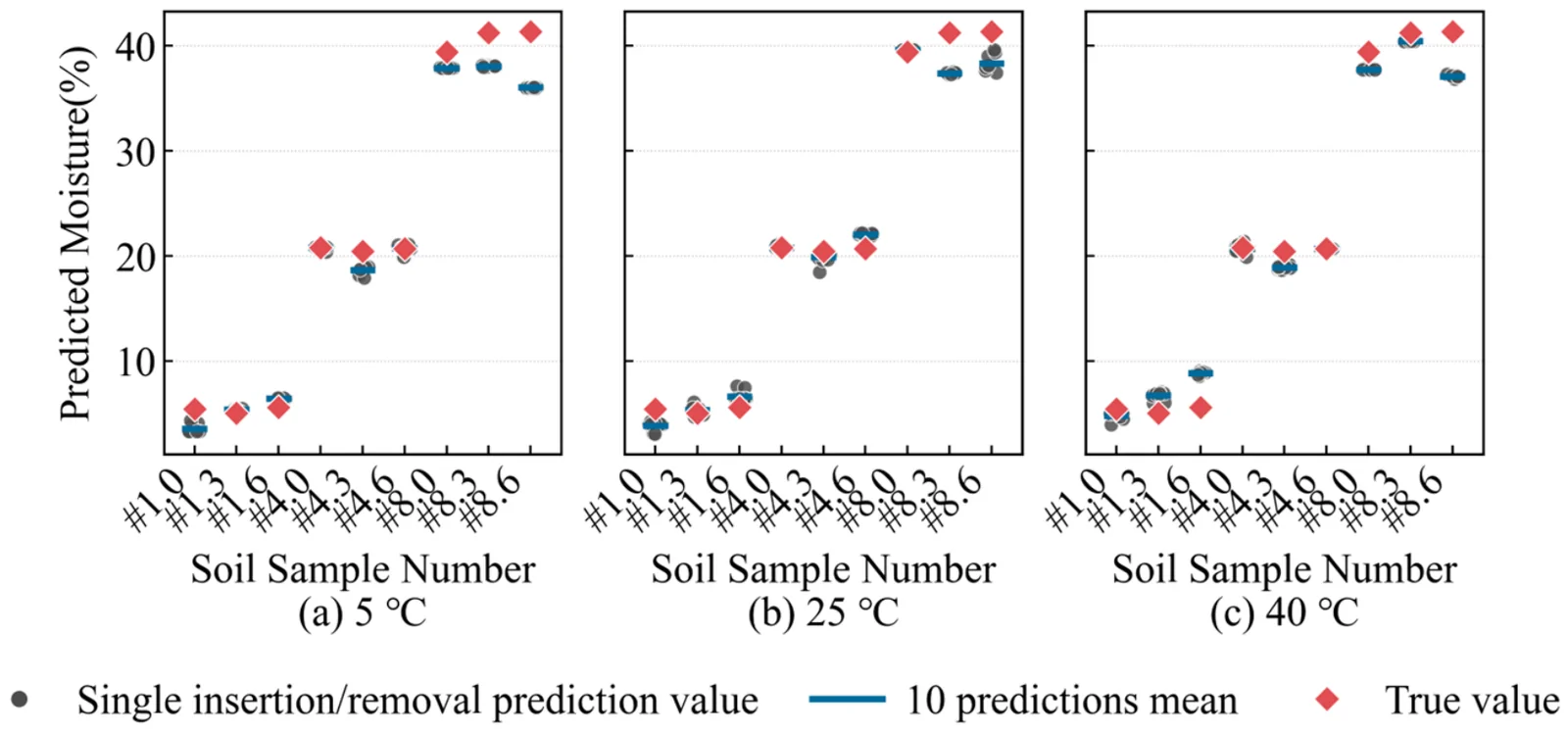
Ten repeated predictions from the same electrode under representative conditions.

Figure 10 plots each reinsertion prediction, condition mean, and reference value. Most repeats clustered closely around their means, indicating limited reinsertion dispersion, although mean bias remained for some low-water content and high-water content/high-salinity samples. The figure, therefore, characterizes repeatability; absolute accuracy should be interpreted with Section 4.1.

### 4.4. Short-Term Stability and Failure Conditions

Short-term stability was tested for #1.0 (5 °C), #4.3 (25 °C), and #8.6 (40 °C) over two consecutive days. The respective mean errors were −2.271 and −2.065, −0.670 and −0.668, and −5.229 and −4.518 percentage points, giving interday differences of 0.206, 0.002, and 0.711 percentage points (Table 12).

**Figure 11:**
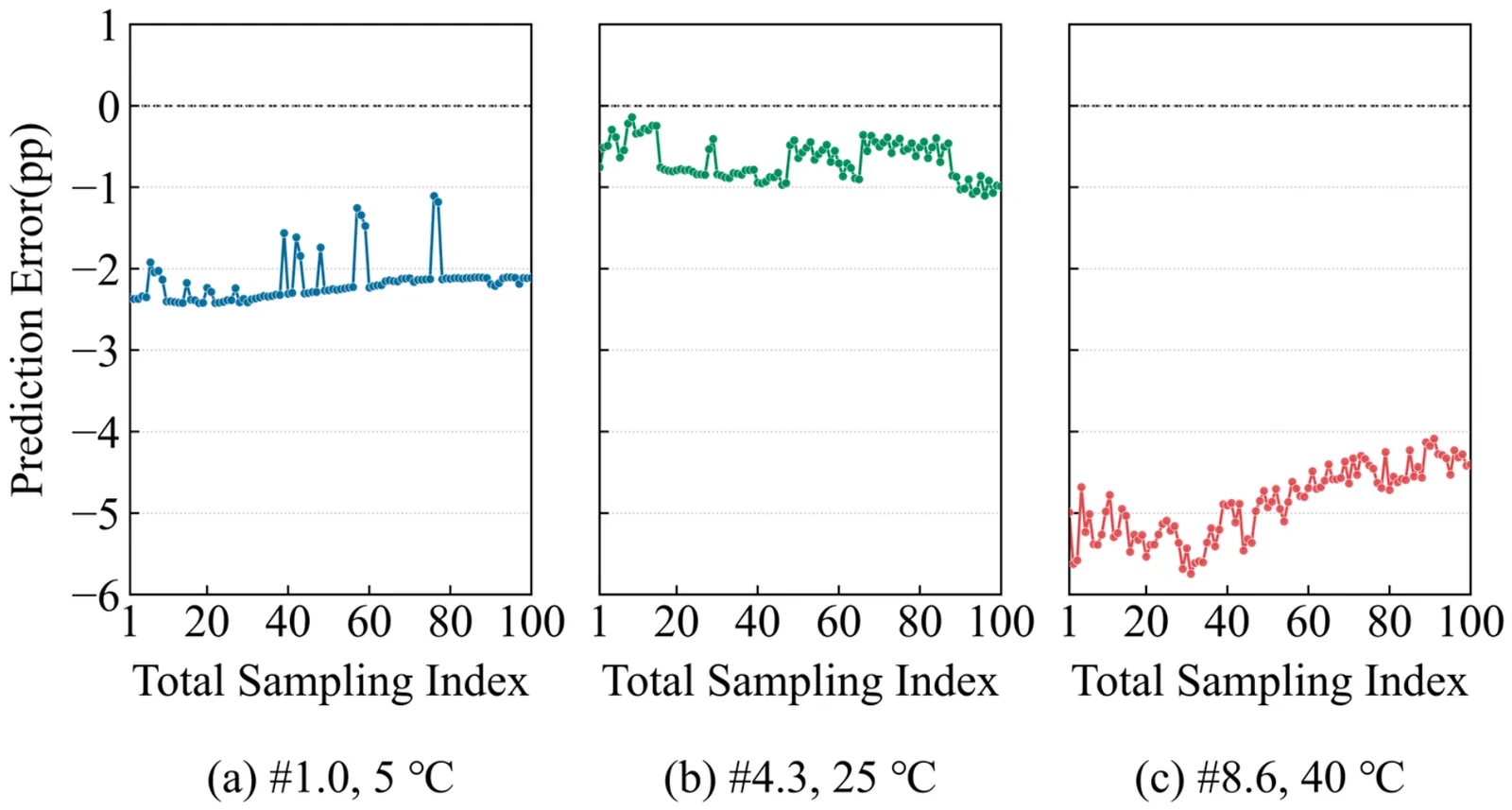
Short-term stability error sequences for three representative conditions.

Within-day standard deviations were 0.205–0.274 percentage points. Under combined high water content, salinity, and temperature, #8.6 retained an approximately 5 percentage point negative bias (Figure 11), identifying a failure condition within the current calibration boundary.

### 4.5. Comparison with a Commercial Sensor

To compare measurement results within the same prepared sandy loam system, a Xingyi CSF11-60 FDR soil moisture sensor (Xingyi Sensor Manufacturing Co., Ltd., Langfang, China) was selected as a commercial reference. According to the product manual, the sensor uses the FDR principle and has a moisture range of 0–60%. The manual further states that the default 3% accuracy is based on calibration in Anhui loam and recommends soil-specific calibration when an accuracy within 3% is required in other soils. The proposed sensor used the soil-specific, parameter-locked multiparameter calibration model developed for the present sandy loam system. CSF11-60 was not recalibrated for this soil; its directly reported volumetric water content values under the manufacturer’s factory calibration were used. Because the calibration states were not matched, this experiment compares the outputs of two complete measurement systems under their respective calibration configurations; it is not a hardware-only comparison.

The same commercial sensor model appeared in our earlier work [6], but all CSF11-60 values analyzed here were newly measured for the present 72-condition experiment.

The comparison used eight volumetric water content levels, three salinity levels, and temperatures of 5, 25, and 40 °C, yielding 72 conditions. Each output was compared with the reference volumetric water content of the corresponding sample. The evaluation metrics were *R*^2^, RMSE, MAE, and bias, where bias was defined as the sensor reading minus the reference volumetric water content. RMSE, MAE, and bias are expressed in percentage points (pp), whereas *R*^2^ is dimensionless.

**Figure 12:**
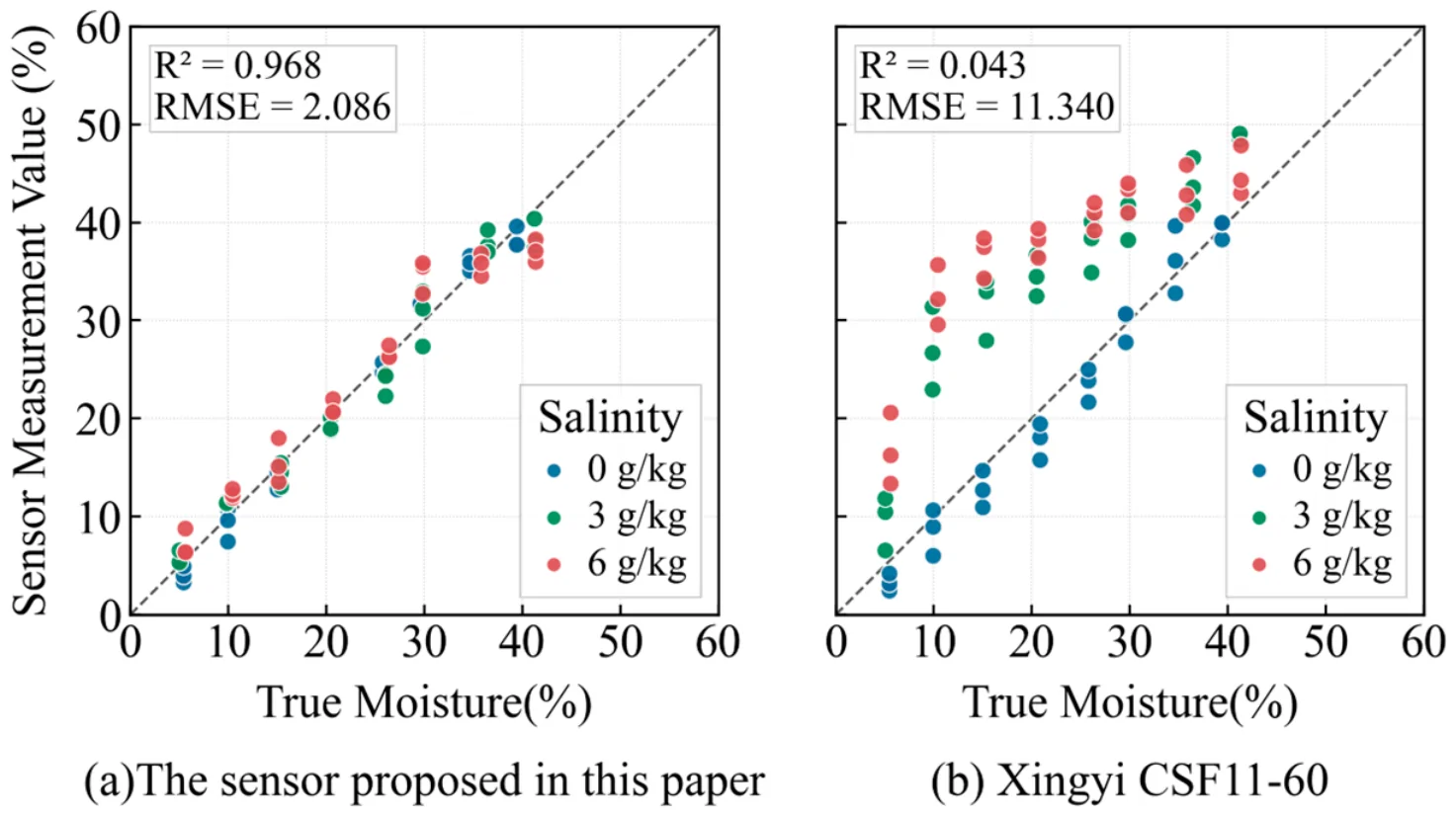
Outputs of the proposed sensor and Xingyi CSF11-60 under their respective calibration states versus reference volumetric water content.

Table 13 reports the results for all 72 shared conditions and for subsets grouped by salinity. Figure 12 compares the outputs of both systems with the reference values. Colors identify salinity levels, and the dashed line is the 1:1 reference line. Measurements closer to this line are closer to the reference volumetric water content under the corresponding condition.

As shown in Table 13 and Figure 12, across all 72 conditions, the proposed sensor achieved *R*^2^ = 0.968, RMSE = 2.086 percentage points, MAE = 1.582 percentage points, and bias = 0.075 percentage points. The factory-calibrated CSF11-60 output yielded *R*^2^ = 0.043, RMSE = 11.340 percentage points, MAE = 9.018 percentage points, and bias = 7.956 percentage points. In Figure 12, most CSF11-60 measurements at salinities of 3 and 6 g/kg lie above the 1:1 line, indicating overestimation in these subsets. Its RMSE values at 0, 3, and 6 g/kg were 2.464, 12.148, and 15.237 percentage points, respectively. At salinity levels of 3 and 6 g/kg, CSF11-60 consistently overestimated soil moisture. Because all errors were positive, the MAE and bias were identical, at 11.186 and 13.843 percentage points, respectively. Under the unequal calibration states used here, the soil-specific multiparameter system had smaller errors than the factory-calibrated CSF11-60 output in this sandy loam system. This result does not establish intrinsic superiority of the proposed sensing hardware. A hardware-focused comparison would require both sensors to be calibrated under the same protocol and soil conditions.

## 5. Discussion

The single nested run, repeated-seed comparison, and deployed measurements answer different questions. Single-run RMSE values were 3.500 and 3.493 percentage points for BPNN and WOA-BPNN, but the 0.007 percentage point difference was not reproduced across 10 seeds; standard BPNN had lower mean RMSE and tail errors. Because WOA adds only offline optimization, these results support standard BPNN as the default model and retain WOA as a secondary analysis.

The measurements in Section 4 were collected before the repeated-seed reassessment using the frozen WOA-initialized BPNN prototype. They are retained as validation of that implemented system, not as evidence that WOA improves predictive accuracy. They also have a different statistical meaning from the outer grouped validation in Section 3: the prototype was trained on all development records, the 72 conditions belonged to the same prepared sandy loam system, and each condition was represented by the mean of 10 readings over a narrower temperature range. The higher *R*^2^ and lower RMSE in Section 4, therefore, do not establish stronger generalization to unseen physical soil samples.

Selective solder mask coverage, time division switching, and the calibration model address different parts of the interference problem. The green solder mask reduces direct conductive contact with the serpentine resistance segment, but no additional coating, potting, or waterproof encapsulation was used. Sequential switching reduces circuit-to-circuit interference. The cross-sensitivity analysis showed that temperature explained most *V_T_* variation, but the moisture-related shift at 5 °C prevents *V_T_* from being interpreted as a pure thermometric signal. The joint model, therefore, uses *V_T_* as a temperature-sensitive proxy together with the resonant frequency and conductivity-sensitive voltage. This interpretation is consistent with the high within-electrode repeatability but does not imply that different electrodes are unconditionally interchangeable.

The reference water content in the development experiment was measured before the temperature series and was, therefore, an initial value rather than a continuously verified value. Keeping the lid closed during equilibration and limiting each opening to approximately 5 min reduced exposure to ambient air. However, the samples were not weighed or oven-dried again after the temperature sequence. Cumulative water loss and moisture redistribution, therefore, cannot be quantified or completely excluded, and they may have introduced uncertainty into comparisons across temperature points. This is a limitation of the present experimental design.

The commercial sensor comparison has the same calibration limitation. The CSF11-60 factory calibration was developed for Anhui loam, whereas the proposed model was fitted to the present sandy loam system. The positive CSF11-60 errors at 3 and 6 g/kg may, therefore, reflect both salinity-related response and transfer of the factory calibration to a different soil. The comparison is useful as an application-level assessment of the two complete systems in the tested conditions, but it cannot separate calibration effects from intrinsic hardware effects.

In the post-deployment repeat measurements, RMSE increased from 1.374 percentage points at 0 g/kg to 2.692 percentage points at 6 g/kg. Together with the smaller high-salinity *V_EC_*–EC1:5 slopes in 63 of the 64 water content–temperature combinations, this result suggests that the reduced VEC response may limit the model’s ability to distinguish among high-salinity states. This reduced discrimination may contribute to the larger errors, but the present data do not establish it as the only cause. Future work should sample the upper salinity range more densely, evaluate independently prepared high-salinity samples, and compare piecewise calibration or boundary-weighted loss functions.

The study used one sandy loam soil, 5–40 °C, 0–6 g/kg salinity, and mainly chamber tests in the same prepared system. Effects of texture, bulk density, mineral composition, and long-term field variation remain untested, and high salinity still produced larger errors and bias. Priority directions for future work include external validation across multiple soil textures and independently prepared sample batches, long-term observations at multiple field sites and across seasons, and control of composite-electrode manufacturing and installation consistency. These are plans for subsequent work; the present manuscript does not report experiments that have not been completed.

Changes in soil texture, mineral composition, dry bulk density, and pore structure can alter dielectric polarization, water distribution, and ionic conduction [8,31]. Consequently, the mapping between the three sensor outputs and volumetric water content may differ in soils outside the present calibration domain. External validation or soil-specific recalibration is, therefore, required before broader application.

Relative to our earlier sensor studies [6,7], the evidence presented here should be interpreted as an extension from single-response FDR sensing to co-located, time multiplexed three-response calibration. The earlier electrode design and FDR conditioning circuit remain part of the platform; the present results evaluate the added conductivity-sensitive and temperature-sensitive channels, their joint calibration, and the embedded implementation. Likewise, reuse of the CSF11-60 model provides continuity in the comparator, whereas the current comparison is based on newly prepared samples and newly acquired measurements. This distinction prevents inherited hardware from being counted again as a novel contribution.

## 6. Conclusions

This study addresses the joint effects of temperature and salinity on FDR resonant frequency, which cannot be adequately covered by fixed single-factor calibration, by developing a soil water content measurement system that combines time division multiplexed three-parameter hardware with a joint three-parameter calibration model. At the same sensing location, the composite electrode sequentially outputs resonant frequency *f*, conductivity-sensitive voltage *V_EC_*, and temperature-sensitive voltage *V_T_*. Among five candidate models, BPNN provided the best overall performance. In the paired 10-seed analysis, BPNN had a lower mean RMSE than WOA-BPNN, so WOA initialization was not supported as a stable accuracy improvement and was retained only as a secondary analysis. Input ablation showed that the conductivity-sensitive voltage provided most of the improvement beyond resonant frequency, whereas the temperature-sensitive voltage provided a smaller improvement in average error metrics. For the frozen WOA-initialized BPNN prototype deployed before the repeated-seed reassessment, repeat measurements under 72 conditions within the same prepared sandy loam system yielded an RMSE of 2.086 percentage points. Within the tested calibration domain (one sandy loam soil, temperatures of 5–40 °C, salinities of 0–6 g/kg, and eight water content levels), the three-parameter calibration model reduced water content errors associated with temperature and salinity variation. The Section 4 measurements validate repeatability of the locked prototype within the same prepared sandy loam system; they do not demonstrate generalization across other soil textures, field sites, independently prepared sample batches, or seasons. External validation in these settings and further assessment of high-salinity error and between-electrode interchangeability are required.

## Figures and Tables

**Table 1:** Three sensing channels and their time division multiplexed states.

Channel	Sensed State	Model Input	Key Components	Closed Switches
FDR channel	Soil water content	MCU-recorded divided frequency *f_MCU_*	MC1648, 2SC3356, MB504, SN74HC393D	S2, S5, S6
Conductivity-sensitive channel	Electrical conductivity	Voltage *V_EC_*	AD9833, Howland current source, AD623, AD736, ADS1110	S1, S4, S7, S9
Temperature-sensitive channel	Local temperature	Voltage *V_T_*	PCB copper resistor, Wheatstone bridge, AD623, ADS1110	S3, S8

**Table 2:** Experimental design of the three-parameter calibration dataset.

Design Factor	Level or Quantity	Implementation Details
Soil	One sandy loam soil	Qingjiangpu District, Huai’an, Jiangsu Province; oven-dried at 105 °C for 24 h; sieved to 2 mm
Dry bulk density	1.522 g/cm^3^	Prepared sandy loam soil used for calibration and validation
Physical soil samples	57	56 water content salinity samples + 1 blank control
Volumetric water content	Eight levels	Reference measured by oven drying at 105 °C for 24 h
NaCl salinity	0–6 g/kg	Seven salinity levels; blank control counted separately
Temperature	5–40 °C	5 °C intervals, eight levels
Dataset size	456 conditions/4560 records	Each sample × 8 temperatures × 10 readings per temperature

**Table 3:** Variation range of resonant frequency at representative volumetric water contents.

Volumetric Water Content Level (%)	Maximum Resonant Frequency (kHz)	Condition of Maximum	Minimum Resonant Frequency (kHz)	Condition of Minimum	Frequency Range (Hz)
5	0.862746	#1.0 0 g/kg 5 °C	0.836309	#1.6 6 g/kg 25 °C	26.437
20	0.842531	#4.0 0 g/kg 15 °C	0.770208	#4.5 5 g/kg 25 °C	72.323
40	0.790514	#8.0 0 g/kg 30 °C	0.708592	#8.5 5 g/kg 35 °C	81.922

**Table 4:** Nested grouped cross-validation and data-processing settings.

Stage	Setting or Scale	Implementation Details
Valid records	4560	All three inputs, reference water content, and data source fields were valid
Physical soil samples	57	Each sample contained 8 temperatures × 10 readings per temperature, for 80 records
Outer split	Stratified grouped five-fold	Grouped by physical soil sample and stratified by water content level; random seed 42
Inner selection	Another five-fold split within each outer training set	Candidate hyperparameters and training epochs were selected only by inner validation RMSE; the outer validation fold was not used for selection
Standardization	Fitted independently on each training fold	Means and standard deviations excluded validation and outer test data
Input/output	Three inputs and one output	[*f*, *V_EC_*, *V_T_*] mapped to oven-drying volumetric water content

**Table 5:** Outer grouped five-fold validation performance of five calibration models.

Model	*R* ^2^	RMSE (pp)	MAE (pp)	Bias (pp)	P95 (|e|) (pp)	MaxAE (pp)
BPNN	**0.911**	**3.500**	**2.761**	0.286	**6.575**	16.152
RF	0.847	4.601	3.718	−0.286	9.535	15.263
XGBoost	0.864	4.341	3.467	−0.092	8.597	**15.251**
Polynomial regression	0.878	4.108	3.330	**−0.067**	7.984	15.727
SVR	0.408	9.045	6.573	0.081	19.661	59.405

Note: Values were calculated by pooling the 4560 predictions from the five outer validation folds. Bold type indicates the best value for each metric; the smallest absolute value is preferred for bias. “(pp)” represents the percentage points of volumetric water content.

**Table 6:** Input-parameter ablation of BPNN under the same nested grouped five-fold validation protocol.

Input Combination	*R* ^2^	RMSE (pp)	RMSE Reduction vs. f (%)	MAE (pp)	Bias (pp)	P95 (|e|) (pp)	MaxAE (pp)
*f*	0.763	5.722	-	4.482	−0.957	11.548	**14.311**
*f* + *V_EC_*	0.906	3.598	37.1	2.876	**−0.147**	6.806	15.843
*f* + *V_T_*	0.779	5.526	3.4	4.441	−0.349	10.926	16.383
*f* + *V_T_* + *V_EC_*	**0.911**	**3.500**	**38.8**	**2.761**	0.286	**6.575**	16.152

Note: All input configurations used the same outer folds, fold-wise preprocessing, nested grouped cross-validation procedure, hyperparameter search space, and random seed. “RMSE reduction vs. f” denotes the relative reduction compared with the frequency-only model. Bold type indicates the best value for each metric; the smallest absolute value is preferred for bias. “pp” represents percentage points of volumetric water content.

**Table 7:** Multivariable analysis of the temperature-sensitive voltage using 456 condition means.

Predictor and Scale	Coefficient	Cluster Bootstrap 95% CI	Standardized Coefficient	Unique ∆*R*^2^
Temperature, per 1 °C	0.004472 V	[0.004385, 0.004560] V	0.987	0.974
Volumetric water content, per 10 pp	−0.000566 V	[−0.001752, 0.000413] V	−0.010	1 × 10^−4^
NaCl salinity, per 1 g/kg	0.000039 V	[−0.000584, 0.000659] V	0.001	1.46 × 10^−6^

Note: The 10 consecutive readings from each temperature–soil sample condition were averaged before analysis. The 95% confidence intervals were obtained from 4000 bootstrap resamples at the physical soil sample level. The full additive model had *R*^2^ = 0.974. “pp” denotes percentage points of volumetric water content.

**Table 8:** Paired repeated-seed comparison of BPNN and WOA-BPNN under a fixed training configuration.

Model	*R* ^2^	RMSE (pp)	MAE (pp)	Bias (pp)	P95 (|e|) (pp)	MaxAE (pp)
BPNN	0.9099 ± 0.0016	3.528 ± 0.031	2.771 ± 0.034	0.133 ± 0.092	6.841 ± 0.108	16.783 ± 0.604
WOA-BPNN	0.9041 ± 0.0033	3.639 ± 0.063	2.875 ± 0.050	0.192 ± 0.123	7.098 ± 0.230	17.802 ± 1.035

Note: Values are the mean ± standard deviation across the 10 paired random seeds. The same outer folds and preprocessing were used for both models at each seed. |Bias| is the absolute systematic bias. “pp” denotes percentage points of volumetric water content.

**Table 9:** Overall and grouped performance of the sensor repeat measurements.

Group	Level	*R* ^2^	RMSE (pp)	MAE (pp)	Bias (pp)
All conditions	-	0.968	2.086	1.582	0.075
Temperature	5 °C	0.959	2.349	1.815	−0.237
	25 °C	0.971	1.966	1.434	0.086
	40 °C	0.973	1.918	1.497	0.376
Salinity	0 g/kg	0.985	1.374	1.084	−0.159
	3 g/kg	0.972	1.980	1.679	−0.303
	6 g/kg	0.947	2.692	1.982	0.686

Note: “(pp)” represents the percentage points of volumetric water content.

**Table 10:** Agreement assessment for three composite electrodes.

Evaluation Item	Object or Condition	Result
ICC(A,1)	Three electrodes, 27 conditions	0.981
Mean between-electrode standard deviation	27 conditions	1.627 pp
Mean between-electrode coefficient of variation	27 conditions	11.520%
Maximum between-electrode standard deviation	#1.6, 40 °C	4.682 pp
Maximum between-electrode range	#1.6, 40 °C	9.033 pp
A-B: bias [95% limits of agreement]	27 conditions	−1.007 [−6.044, 4.031] pp
A-C: bias [95% limits of agreement]	27 conditions	−0.651 [−5.821, 4.518] pp
B-C: bias [95% limits of agreement]	27 conditions	0.355 [−5.013, 5.723] pp

Note: “(pp)” represents the percentage points of volumetric water content.

**Table 11:** Within-electrode repeatability assessment.

Evaluation Metric	Result	Corresponding Condition
Pooled repeatability standard deviation	0.296 pp	All 27 conditions
Mean condition-specific standard deviation	0.227 pp	All 27 conditions
Mean condition-specific coefficient of variation	2.541%	All 27 conditions
Minimum condition-specific standard deviation	0.009 pp	#8.0, 25 °C
Maximum condition-specific standard deviation	0.736 pp	#8.6, 25 °C
Maximum repeatability limit r	2.061 pp	#8.6, 25 °C

Note: “(pp)” represents the percentage points of volumetric water content.

**Table 12:** Two-day short-term stability under representative conditions.

Condition	Day 1 Mean Error (pp)	Day 2 Mean Error (pp)	Day 1 Standard Deviation (pp)	Day 2 Standard Deviation (pp)	Interday Mean Difference (pp)
#1.0 (5 °C)	−2.271	−2.065	0.205	0.274	0.206
#4.3 (25 °C)	−0.670	−0.668	0.241	0.226	0.002
#8.6 (40 °C)	−5.229	−4.518	0.267	0.220	0.711

**Table 13:** Application-level volumetric water content outputs of the proposed sensor and the Xingyi CSF11-60 under their respective calibration states.

Sensor	Condition	*R* ^2^	RMSE (pp)	MAE (pp)	Bias (pp)
Proposed sensor	All conditions	0.968	2.086	1.582	0.075
CSF11-60		0.043	11.340	9.018	7.956
Proposed sensor	0 g/kg	0.985	1.374	1.084	−0.159
CSF11-60		0.952	2.464	2.025	−1.159
Proposed sensor	3 g/kg	0.972	1.980	1.679	−0.303
CSF11-60		−0.044	12.148	11.186	11.186
Proposed sensor	6 g/kg	0.947	2.692	1.982	0.686
CSF11-60		−0.707	15.237	13.843	13.843

Note: The proposed sensor used a calibration model developed for the present sandy loam system. The CSF11-60 values are factory-calibrated default outputs without soil-specific recalibration. The comparison, therefore, concerns complete-system outputs under these calibration states and does not isolate hardware performance. “(pp)” represents the percentage points of volumetric water content.

## Data Availability

The raw data supporting the conclusions of this article will be made available by the authors upon request.
